# LDPCD: A Novel Method for Locally Differentially Private Community Detection

**DOI:** 10.1155/2022/4080047

**Published:** 2022-01-10

**Authors:** Zhejian Zhang

**Affiliations:** College of Computer Science, Chongqing University, Chongqing 400044, China

## Abstract

As one of the cores of data analysis in large social networks, community detection has become a hot research topic in recent years. However, user's real social relationship may be at risk of privacy leakage and threatened by inference attacks because of the semitrusted server. As a result, community detection in social graphs under local differential privacy has gradually aroused the interest of industry and academia. On the one hand, the distortion of user's real data caused by existing privacy-preserving mechanisms can have a serious impact on the mining process of densely connected local graph structure, resulting in low utility of the final community division. On the other hand, private community detection requires to use the results of multiple user-server interactions to adjust user's partition, which inevitably leads to excessive allocation of privacy budget and large error of perturbed data. For these reasons, a new community detection method based on the local differential privacy model (named LDPCD) is proposed in this paper. Due to the introduction of truncated Laplace mechanism, the accuracy of user perturbation data is improved. In addition, the community divisive algorithm based on extremal optimization (EO) is also reﬁned to reduce the number of interactions between users and the server. Thus, the total privacy overhead is reduced and strong privacy protection is guaranteed. Finally, LDPCD is applied in two commonly used real-world datasets, and its advantage is experimentally validated compared with two state-of-the-art methods.

## 1. Introduction

Due to the rapid development of Internet technology, APPs with various functions have brought great convenience to the daily interaction among users. After integration, these relationship data of users can be exploited to build social graphs, from which service providers can mine valuable information such as frequent subgraphs [1, 2], average path length among users [3], and the community structure of users [4–6]. In particular, community structure is an important feature of social graphs. On the one hand, based on network topology architecture and user attributes, various user communities and interest groups will be mined for the futural personalized recommendations [7, 8]. On the other hand, as an important manifestation of topological features, community structure has a signiﬁcant guiding role in creating synthetic social graphs [9]. Therefore, the exploration of community information from social networks has attracted extensive attention in the ﬁeld of academy and industry.

Community detection on the social graph requires the collection of users' social relationships. However, most social links among users are sensitive and private information. If the user uploads such data without reservation or the server does not take any privacy protection measures in the centralized data analysis, the user's local information may be exposed to the risk of leakage and inference attack. For example, in 2018, Facebook was accused of leaking tens of millions of user personal information to the UK-based third-party ﬁrm Cambridge Analytica [10]. This privacy scandal indicates that one of the main problems to be solved in the community mining of social networks is the privacy protection during the collection of user relationship data.

As far as we know, a promising model which can be utilized to resolve the privacy issue posed by the untrusted service provider is local differential privacy (LDP) [11]. LDP is a privacy protection framework inherited from centralized differential privacy (DP or CDP) for privacy protection during data collection. The real data are perturbed by the user on the local terminal and then uploaded to the data curator. Both in industrial production areas and academic research studies [12–18], this model has been widely utilized because of its strong resistance to attack based on any background knowledge and its exclusion of the assumption of a fully trusted server.

At present, community detection based on LDP has become a research hotspot in privacy protection of big data [8, 19, 20]. In this context, the protective measures of social relationship data are transferred from centralized overall processing to distributed processing by each user. This data collection pattern poses a key problem for the community detection task of social graphs. Since most of the community detection algorithms of graph data require that the real and speciﬁc structure of the entire network be known, under the local privacy protection, it is difficult for the server to directly conduct community detection algorithm with false information uploaded by users instead of a true social graph. Moreover, serious damage to the network topology will be posed when the users independently add noise to their local relationship data. This will eventually cause excessive loss of graph structure information and seriously affect the utility of community detection results [19–21]. Therefore, it is speculated that studying the community detection problem of the social graph under LDP is difficult.

This paper intends to respectively modify existing community detection method of graph data and perturbation method of users' local data under LDP. Thus, a novel privacy protection community detection method is designed, which is named LDPCD. In its framework, a community divisive algorithm based on extremal optimization (EO) is used as the basis of the community detection method. When executing the EO algorithm, the total number of queries on the user's degree vector and the times of grouping adjustment will increase sharply with the network scale. Therefore, under a limited total privacy budget, there exists a problem of extremely large errors in the results of a single query. In this regard, the truncated Laplace mechanism is used to limit the disturbance range of the user's degree, thereby reducing the calculation error of the user's ﬁtness value. Appropriate reﬁnements are also made to the existing EO algorithm under the protection of LDP to signiﬁcantly reduce the total interaction times between users and the server. Finally, LDPCD is adopted to conduct community detection on two social network datasets to prove its effectiveness. In summary, the following contributions of this paper are made:

1. )A novel community detection method LDPCD under local differential privacy protection is proposed, which can obtain better community detection results under higher local privacy protection requirements.
2. In order to solve the problem of large error caused by Laplace mechanism, the truncated Laplace mechanism is introduced to optimize the local perturbation of user's degree vector. Moreover, we provide rigorous theoretical proof that the new noise addition method satisﬁes *ε*-LDP.
3. By reﬁning the community divisive algorithm based on extremal optimization, the interaction times between users and the server as well as the total privacy cost are reduced, and the utility of community division is also guaranteed.
4. Through experimental evaluation on two commonly used social network datasets, the community detection results of LDPCD are compared with those of two state-of-the-art methods of graph data analysis under LDP [19, 20] to demonstrate the accuracy and effectiveness of our proposed method.

The rest of the paper is structured as follows.  Section 2 introduces the research status of LDP in graph data analysis.  Section 3 elaborates the preliminary knowledge of community detection and LDP protection on graph data and gives the deﬁnition of the problem in this paper.  Section 4 describes the framework of LDPCD and its implementation details.  Section 5 presents and analyzes the experimental results. Finally, Section 6 draws the conclusion.

## 2. Related Works

In recent years, research studies on protecting the topological characters and user relationships in social graph data under LDP have attracted widespread attention of scholars [8, 19, 20, 22–28]. According to different analysis objects of social graph data, the existing work can be summarized in two aspects, which are statistical graph metrics estimation and synthetic social graph generation.

### 2.1. The Application of LDP in Statistical Graph Metrics Estimation

The statistical metrics of the social graph are important objects of graph data mining. As coarse-grained information, these statistical metrics highly condense certain properties of the social graph, which can express complex topological relationships through simple numerical values. Therefore, some studies adopted LDP mechanisms to perturb user's social data and analyzed many types of statistical graph metrics such as degree distribution [8, 29], clustering coefficient distribution [20, 30], edge weight distribution [31], and modularity [20].

Jacob et al. [23] proposed a method for estimating the frequency of subgraphs based on LDP. The central server aggregates this local statistical information with calibrated noise after interacting with the users to estimate the total number of k-stars and triangles in the entire graph. Sun et al. [25] formulated a stringent deﬁnition of decentralized differential privacy to provide adequate protection for the information of each user and her neighbors. The total frequency of triangles, three-hop paths, and k-clique in the social graph is thus precisely estimated using a noise injection method that satisﬁes this privacy deﬁnition. Wei et al. [8] proposed using LDP in the collection of attribute graph data. After the random-jump perturbation to the user's degree and the randomized response mechanism to the user's binary attribute value, the original degree distribution and the joint distribution of attribute data are, respectively, restored by unbiased estimation and EM algorithm. For the social graph with edge attributes, Liu et al. [24] proposed a novel privacy deﬁnition (attribute-wise LDP) with stronger protection than edge differential privacy. Accordingly, a novel perturbation mechanism was designed to protect all edges with the same attribute for each user. The corresponding method for the restoration of statistical metrics was also proposed to estimate the frequency of nodes with certain attribute edges and the degree-attribute joint distribution of the social graph. Ye et al. [20, 27] argued that, for the estimation of most statistical graph metrics (such as node clustering coefficient and subgraph modularity) under LDP, it is sufficient to only query the noisy degree and adjacent bit vector of each user. Based on this point, they proposed a general framework LFGDPR, which analyzes the optimal allocation scheme of privacy budget to separately perturb the two items and provides the corresponding unbiased estimation algorithm for different graph metrics.

### 2.2. The Application of LDP in Synthetic Social Graph Generation

In the various applications of differential privacy on social graph data, it is a popular but challenging research task to use appropriate graph generation models to generate a synthetic and privacy-guaranteed social graph for its publishing to third parties [22, 32]. With the rise of social graph analysis based on LDP, the research on the synthesis of a private graph based on the user's decentralized perturbed information is also gradually unfolding [8, 19, 28].

Qin et al. [19] conducted pioneering research on this ﬁeld and proposed a graph data collection and synthetic graph generation method under LDP, which is named LDPGen. In more detail, each time LDPGen partitions all users into disjoint groups and queries each user for her perturbed degrees under the grouping, users with similar degree vectors are clustered together to form a new user partition. This process of grouping-inquiry-grouping iterates until the privacy budget is depleted. Based on the ﬁnal partition, a synthetic social graph is generated by using the graph-generating model of Chung-Lu [33] for further analysis. With the same research object as in [19], Zhang et al. [28] proposed to collect users' noisy degrees by means of secure multiparty computation to form several user groups. Then, each user adopts an optimized randomized response scheme to perturb its adjacency vectors in different groups. Finally, the synthetic social graph is generated through the synthesized adjacency matrix after aggregating the perturbed bit vectors of all users. Based on the collected noisy data from all users, Wei et al. [8] used the attribute graph model (AGM) [34] and takes the estimated distribution of degrees and attribute values as input parameters to generate the initial seed graph. In order to preserve the structure and community information of the original graph, the community detection algorithm of CESNA [6] is adopted. Through continuous iterative community detection of the seed graph and the modiﬁcation of the edges and attribute values in it, the convergent synthetic attribute graph with high utility is ﬁnally generated. In addition, Ye et al. [20] proposed the LFGDPR framework that can be applied to the unbiased estimation of the modularity of any subgraph. On this basis, the Louvain community detection algorithm [4] is used to divide users into communities, and a new synthetic social graph is generated based on the community detection results.

In general, the existing work can analyze some commonly used statistical metrics of graph data and generate synthetic social graphs under LDP. However, in some of it, the problem of community detection is mostly presented as a part of the whole research content and closely connected with the final synthetic graphs, which means that the quality of the graphs will have a significant impact on the utility of the community division result. In this paper, we attempt to design a more straightforward method without private graph generation and gradually restore the community structure through multiple user-server interactions.

## 3. Problem Deﬁnition

In this section, we brieﬂy introduce the prerequisite knowledge of community detection and local differential privacy for graph data; then, we give a detailed deﬁnition of the considered problem.  Table 1 describes the meaning of some notations used in this study.

### 3.1. Nonprivate Community Detection in Social Graphs

Many research studies have been conducted on the community detection of social graphs. Most classical methods of community detection are dedicated to optimizing the modularity of the community division of the entire graph. Among them, the EO-based heuristic algorithm has been widely used owing to its high computational efficiency and fast convergence speed [5].

In the method of EO-based community detection, the global variable is the modularity *Q* of a community division of all users, whereas the local variables are the contribution of each node to the total modularity. The contribution *q*_*i*_ of node *i* is as follows:

(1) $$\begin{array}{c} q_{i}=\delta_{c\left(i\right)}-\delta_{i}a_{c\left(i\right)}, \end{array}$$

where *δ*_*c*(*i*)_ represents the number of edges connected between a node *i* belonging to community *c* and the other nodes in the same community, *δ*_*i*_ represents the total degree of node *i*, and *a*_*c*(*i*)_ represents the proportion of the degree sum of all nodes in community *c* to the degree sum of all nodes in the entire network. The relationship between the modularity *Q* as a global variable and the local variable *q*_*i*_ is as follows:

(2) $$\begin{array}{c} Q=\frac{1}{2L}\sum_{i}q_{i}=\sum_{c}\left[\frac{L_{c}}{L}-{\left(\frac{\sum_{i\in c}\delta_{i}}{2L}\right)}^{2}\right], \end{array}$$

where *L*_*c*_ and *L* represent the number of edges in community *c* and the total edges in the entire network, respectively. Since the value range of *Q* is [−1/2, 1], to maintain the consistency, the local variable is normalized as *λ*_*i*_ with the same range, which is deﬁned as the ﬁtness of user *i*:

(3) $$\begin{array}{c} \lambda_{i}=\frac{\delta_{c\left(i\right)}}{\delta_{i}}-a_{c\left(i\right)}. \end{array}$$

Therefore, the greater the ﬁtness value of a node, the greater its contribution to the modularity of the community structure.

After deﬁning the ﬁtness of each user, the heuristic divisive algorithm of EO can be described as follows:

1. Initialization: the entire network is randomly divided into two groups, each of which has the same number of nodes. This is regarded as the initial community structure of the network.
2. Iteration: in each iteration, after the ﬁtness values of all users have been calculated and sorted, the node with the lowest ﬁtness is considered to contribute the least to the modularity of current bipartition of users and is moved to the other group. After each move, calculating the new two-dimensional degree vector of all users based on the original graph and updating their ﬁtness accordingly is necessary.

By repeating step (2), an optimal bipartition state will be ﬁnally obtained. In addition, its modularity *Q*_b_ (also defined as bipartition modularity) reaches a locally optimal value and no longer increases. Afterward, all edges between the two resulting groups are removed, and the abovementioned initialization grouping and iteration process is independently continued in each subgraph formed by the ﬁnal groups (each with their own *Q*_b_ when divided into two parts), thereby further splitting the users' community.

### 3.2. Threat Model

The community detection algorithm described in this paper involves multiple rounds of interaction between two participants, i.e., the user and the server. The user is considered to be trusted because she only keeps her social relationship data locally. However, the server is considered semitrusted. On the one hand, the central server collects true relationship data uploaded by users and reconstructs the real social graph to provide users with personalized services based on the mining results of it. On the other hand, the users' real data may be disclosed to other untrusted third parties. In addition, even if a user only uploads the true statistical values of some coarse-grained information (such as user's degree), the central server will infer whether there is a social link between this user and another targeted user based on his existing background knowledge or true information provided from other users in collusion.

### 3.3. Privacy Deﬁnition

Based on the threat model described in Section 3.2, to collect user's private social relationships without relying on a trusted server, we should resort to LDP mechanisms for the protection of each user's local and limited information.

In the graph data analysis of LDP, privacy deﬁnitions are generally divided into the node LDP and edge LDP [19]. In our scenario, whether two targeted users have a friendly relationship is considered private information to be protected, which coincides with the deﬁnition of edge LDP. Considering that any edge in a social graph can affect at most one bit of each user's neighbor list, edge LDP is deﬁned as follows:

**Definition 1 (edge LDP, see [19]).**

For a social network with *N* user nodes, a randomized mechanism *M* deﬁned on {0,1}^*N*^ satisﬁes *ε*-edge LDP if any user *i* and her two neighbor lists *l*_*i*_, *l*_*i*_′ differ only in one bit, as well as any possible output subset *S* ∈ Range(*M*). The following probability inequality holds:

(4) $$\begin{array}{c} \mathrm{Pr}\left[M\left(l_{i}\right)\in S\right]\leq e^{\varepsilon }\mathrm{Pr}\left[M\left(l_{i}^{\prime }\right)\in S\right]. \end{array}$$

Regarding the EO-based community detection algorithm adopted in this paper, the calculation of user ﬁtness mainly involves the user's degree vector for a certain grouping situation. Thus, we adopt the degree perturbation mechanism proposed in [19]. In particular, the central server divides all users into *k* groups, denoted as *ξ*={*U*_1_,…, *U*_*k*_}. After the grouping information is distributed to all users, each user tallies her degree in each group and obtains the corresponding degree vector **δ**_*i*_={*δ*_*i*_^1^,…, *δ*_*i*_^*k*^}. Because the presence or absence of an edge in the social graph will affect at most one degree value in the degree vector of each user by 1, the user adds independent Laplace noise with a mean value of 0 and a scaling parameter of 1/*ε* to each dimension of the degree vector, i.e.,

(5) $$\begin{array}{c} {\tilde{\delta }}_{i}=\left\{{\tilde{\delta }}_{i}^{1},\ldots ,{\tilde{\delta }}_{i}^{k}\right\}=\left\{\delta_{i}^{1}+\text{Lap}\left(\frac{1}{\varepsilon }\right),\ldots ,\delta_{i}^{k}+\text{Lap}\left(\frac{1}{\varepsilon }\right)\right\}. \end{array}$$

Therefore, if the neighboring degree vectors **δ**_*i*_ and **δ**_*j*_ satisfy *δ*_*i*_^*r*^=*δ*_*j*_^*r*^ for *r* ∈ [1, *k*] and *r* ≠ *m*, as well as |*δ*_*i*_^*m*^ − *δ*_*j*_^*m*^|=1, then, for any possible output result **s**=(*s*_1_,…, *s*_*k*_) ∈ Range(*M*), there is

(6) $$\begin{array}{c} \frac{\mathrm{Pr}\left[M\left(\delta_{i}\right)=s\right]}{\mathrm{Pr}\left[M\left(\delta_{j}\right)=s\right]}=\frac{\mathrm{Pr}\left[{\tilde{\delta }}_{i}^{1}=s_{1}\right],\ldots ,\mathrm{Pr}\left[{\tilde{\delta }}_{i}^{k}=s_{k}\right]}{\mathrm{Pr}\left[{\tilde{\delta }}_{j}^{1}=s_{1}\right],\ldots ,\mathrm{Pr}\left[{\tilde{\delta }}_{j}^{k}=s_{k}\right]}=\frac{\mathrm{Pr}\left[{\tilde{\delta }}_{i}^{m}=s_{m}\right]}{\mathrm{Pr}\left[{\tilde{\delta }}_{j}^{m}=s_{m}\right]}\leq e^{\varepsilon }. \end{array}$$

This shows that the degree vector perturbation mechanism satisﬁes *ε*-edge LDP.

In addition, considering our community detection scenario, the user and the server interact several times in the iterative process of ﬁtness calculation and grouping adjustment. Since any relationship edge of one user affects the degree results for each query of the server to the greatest extent, according to the sequential composition property of DP [35], in the entire process of community detection, the total privacy cost for each user is equal to the sum of all the privacy budget consumed by her interactions with the server.

### 3.4. Accuracy Deﬁnition

The community detection on a real social network can get the community division of users close to the facts. In this study, we use the community detection result of a real social network based on the EO algorithm as the ground truth, denoted by **C**_t_. The result of community detection under LDP is expressed as **C**_p_. For a certain deviation between **C**_p_ and **C**_t_, the accuracy of privacy-preserving community detection is measured using three metrics: modularity, ARI, and AMI. These three metrics are described in detail in Section 5.1.3.

### 3.5. Problem Statement

In this study, we aim to ﬁnd a tradeoff between the privacy protection of user social relationship data and the utility of community detection results. Based on the deﬁnitions of privacy and accuracy given in Sections 3.3 and 3.4, as well as the EO-based community detection method, in this section, we formally describe the problem of the community detection for social networks under LDP guarantees.

**Definition 2 (community detection for social networks under LDP).**

In this study, LDPCD, a novel framework, is designed for community detection on social networks under LDP.

1. The framework guarantees that any relationship data of each user satisﬁes *ε*-differential privacy for the other users and the server
2. Based on the EO algorithm, the framework exploits the degree vector information uploaded by users and uses a community divisive algorithm to divide users into several communities
3. The framework improves the existing classical data perturbation mechanism to ensure that the results of community detection can achieve reasonable utility under strong privacy protection

Studying community detection under LDP protection is challenging because of two main reasons. First, the central server cannot use agglomerative algorithms to cluster users into communities, which is because these algorithms cannot be implemented by correctly inferring the truthfulness of the perturbed links in the local region with high probability, thereby affecting the fusion process of nodes and communities based on the greedy algorithm. Second, in the context of LDP, each user interacts with the server several times to continuously optimize the community division results. For a moderate total privacy budget, overly small distributed budget may result in the utility of community division to not signiﬁcantly improve with the increase in the number of interactions. Therefore, in response to the ﬁrst one, the community divisive algorithm is used as our basic method, and user statistics such as the degree vector are exploited to guide the division and adjustment of user communities. To mitigate the large perturbation error caused by excessive interactions, the conventional data perturbation mechanism is improved to enhance the accuracy of community detection under reasonable privacy protection strength.

## 4. Methods

In this section, we propose LDPCD, a novel framework, for community detection of social graphs under LDP protection. First, this framework is described in detail. Then, the drawbacks of directly applying the existing noise injection method of LDP to the EO-based community detection algorithm are analyzed. Finally, a modiﬁed data perturbation satisfying LDP and a reﬁned EO-based community detection algorithm are proposed.

### 4.1. Framework

**Figure 1:**
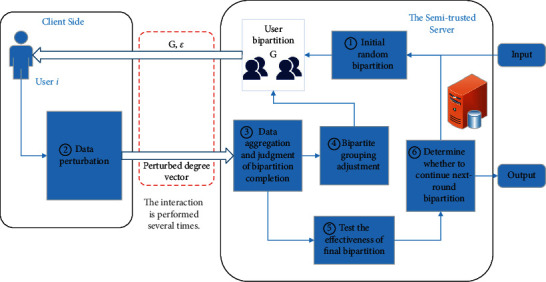
The framework of LDPCD.

As shown in Figure 1, LDPCD mainly comprises two building blocks: the multiple interactions between users and the server and the server-side iterative processes of the generation of user communities. The user-side operation mainly comprises the calculation and local perturbation of the degree vector. The iterative processes comprise the multiple degree queries and iterative adjustment of user's bipartition until the bipartition modularity converges, and the iterative optimal bipartition of user's community until the user partition is stable.

At the user side, user *i* receives the bipartite grouping **G** from the server, generates her true degree vector **δ**_*i*_, and perturbs it according to the assigned privacy budget *ε* and then uploads the perturbed degree vector ${\tilde{\delta }}_{i}$ (step ②).

At the server side, the overall data operation process is shown in steps ① and ③ to ⑥ in Figure 1. In the input step, the server gets the ID information of all surveyed users and forms a user set **U**_0_. Meanwhile, a community division **C**_0_ is initialized, where **C**_0_={**U**_0_}. Next, the server performs multiple rounds of bipartition to divide all users into several communities. In particular, when the bipartition process reaches the *r*th round, the server starts from the user community division result of the previous round and initializes the *r*th round's community as **C**_*r*_=∅, then sets each subset in **C**_*r*−1_ as an independent user community **U**, and performs the bipartition operation separately.

In the initial case of the bipartition operation, the server side randomly bisects **U** and obtains the initial bipartite grouping **G**={**U**^1^, **U**^2^} (step ①). After querying the perturbed degree vectors of all users (step ②), the server collects them and computes the corresponding ﬁtness for each user in the EO-based algorithm and the bipartition modularity value of the current grouping (step ③). Afterward, the server iteratively sorts the users' fitness to obtain the sequence $\tilde{\lambda }$, adjusts the bipartite grouping according to $\tilde{\lambda }$, and recalculates the fitness of each user until the grouping situation stabilizes (step ④). The stabilized **G** and privacy budget *ε* are distributed by the server to each user in **U** for the next query regarding degree vectors (step ②). After collecting all perturbed data, the server repeats the computation of ﬁtness as well as bipartition modularity and the iterative adjustment of the user's grouping. The adjustment-query-adjustment process for the ﬁnal bipartition result will last until the bipartition modularities obtained by two neighboring queries have a subtle gap, which indicates that the optimal bipartition of **U** is ﬁnally obtained and its final grouping **G**_f_ is thus formed (step ③).

During the *r*th round of user community division, after judging that the optimal bipartition of each subset **U**_*r*−1_ ∈ **C**_*r*−1_ is completed (step ③), checking whether its **G**_f_ can cause an increase in the total modularity $\tilde{Q}$ compared to itself without bipartition (step ⑤) is necessary. If the gain is greater than a certain expected error, **C**_*r*_**=C**_*r*_ ∪ **G**_f_ is computed to update the *r*th round's community division. Otherwise, **C**_*r*_**=C**_*r*_ ∪ {**U**_*r*−1_} is executed, indicating that any bipartition cannot pose an obvious gain in $\tilde{Q}$. Whether to perform a new round of community bipartition is decided based on the comparison result of **C**_*r*−1_ with **C**_*r*_ (step ⑥). Thus, in the entire process of the community divisive algorithm based on EO, the server side iteratively performs the optimal bipartition of user subsets (also as communities) for several rounds, which start from the ﬁrst round of community bipartition on all users and ﬁnally ends when the user community divisions of two neighboring rounds are identical, and the total modularity stabilizes to get the ﬁnal community detection result of all surveyed users.

### 4.2. A Naive Method

Since Laplace mechanism is commonly used for differential privacy protection of numerical data, an intuitive approach is to inject Laplace noise in user's true degree vector (as mentioned in Section 3.3). After making minor adjustments to user groups based on user perturbation data, the server needs to inform all users within the bipartite grouping about the migrated users, thus making all users update their degree vectors according to the new grouping situation. On this basis, we refer to the classical EO algorithm [5] in our naive method and only move the user with the lowest ﬁtness to the other group in each grouping adjustment and continue the query for the user's updated perturbed degree vectors. As we can imagine, the migration of one user only results in a slight change in the degree vectors of some users. However, determining whether and to what extent the degree vector of the other nodes that have not been moved have changed is impossible for the server because he has no access to the true social graph of the surveyed users under LDP. Therefore, each time of user grouping adjustment has to be allocated some privacy budget for querying about the updated degree vectors.

#### 4.2.1. Problems of the Naive Method

In the abovementioned naive method, the conventional Laplace mechanism is used for data perturbation. The server executes the iterative process of the classical EO algorithm by multiple interactions with users and using the perturbed data for ﬁtness calculation and node migration. Two main problems are encountered in this process. On the one hand, with excessively small privacy budget distributed for each query, the addition of Laplace noise can considerably distort the true degree vector when there is no limitation of output range for the noisy degree. The perturbed data with large error will considerably affect the grouping adjustment process, resulting in low modularity and poor utility of the subsequent community division results. On the other hand, the classical EO algorithm requires that every time a single user node changes its group, the information of each user is re-queried, which signiﬁcantly increases communication cost between users and the server. Therefore, applying this naive approach to practical community detection under LDP protection is nearly impossible.

### 4.3. Data Perturbation

Considering the limitations of Laplace mechanism when applied to the EO-based community detection algorithm, the truncated Laplace mechanism [36] which restricts the perturbation range of user's degree is used as our data noising scheme, thereby resolving the problem of the poor utility of the community division result caused by the large error under low privacy budget.

In the Laplace distribution *f*(*x*)=*e*^−|*x* − *μ*|/*σ*^/2*σ*, the output range is the real domain. However, to ensure that the result of user degree is meaningful after perturbation, the output range of the LDP-based mechanism should be limited from 0 to the total number of users or to an even shorter interval of the degree value, which can prevent a small degree from being perturbed under the conventional Laplace mechanism and resulting in a negative one with large absolute value and seriously affecting the estimation accuracy of user ﬁtness. In this regard, the truncated Laplace mechanism [36], as an improved method of Laplace mechanism, truncates the inﬁnite range of perturbation results. Moreover, to ensure that the integral of the output probability density of all values in the truncated range is equal to 1, the probability density function *f*(*x*) is multiplied by a normalization parameter, which guarantees that the true degree results are always output with the maximum probability and meanwhile increases the output probability of all values within the output interval. The integral of probability density function in this interval is 1, whereas the output probability of all values outside this truncated range is 0, thus improving data accuracy after perturbation.

**Figure 2:**
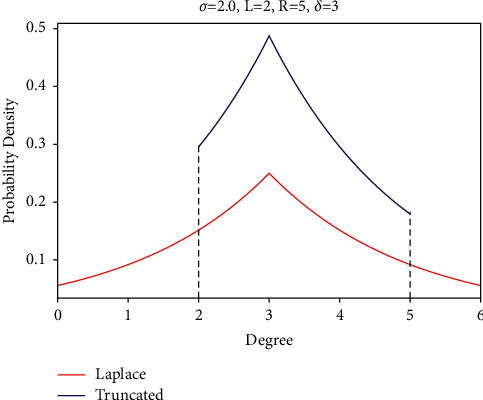
Laplace distribution and truncated Laplace distribution.

As shown in Figure 2, when the users use the truncated Laplace mechanism to perform degree perturbation locally, the limit output range should be determined according to the true value of the user, and it is expressed by (*L*, *R*), where *L*, *R* ∈ *ℕ* (here, the symbols *L* and *R*, respectively, denote the left and right boundary of the output range, and the true degree *δ* satisﬁes *L* < *δ* < *R*). Because the information that users need to protect is the existence or absence of an edge in the true social graph, the sensitivity of each dimension of the local degree vector is Δ*δ*=1. Given the privacy budget *ε* of each time of query, the scaling parameter *σ* in the truncated Laplace distribution function is obtained, i.e.,

(7) $$\begin{array}{c} \sigma =\frac{2\Delta \delta }{\varepsilon }=\frac{2}{\varepsilon }. \end{array}$$

(In Section 4.5.1, we give detailed proof of the value of *σ*). Then, the integral on the intervals (−*∞*, *L*) and (*R*, +*∞*) is calculated according to the probability density function of the Laplace distribution, i.e.,

(8) $$\begin{array}{c} I_{L}=\int_{-\infty }^{L}\frac{1}{2\sigma }e^{-\left|x-\delta \right|/\sigma }\text{d}x=\frac{1}{2}e^{-\left|\delta -L\right|/\sigma }, \end{array}$$

(9) $$\begin{array}{c} I_{R}=\int_{R}^{+\infty }\frac{1}{2\sigma }e^{-\left|x-\delta \right|/\sigma }\text{d}x=\frac{1}{2}e^{-\left|\delta -R\right|/\sigma }. \end{array}$$

To make the integral of the probability density function on the truncated range equal to 1, the output probability density *f*(*x*) of any real number *x* ∈ [*L*, *R*] is multiplied by the normalization coefficient *n*_*δ*_, which is calculated according to *n*_*δ*_=1/(1 − *I*_*L*_ − *I*_*R*_), and a normalized probability density *p*(*x*) is obtained, i.e.,

(10) $$\begin{array}{c} p\left(x|x\in \left[L,R\right]\right)=\frac{n_{\delta }}{2\sigma }e^{-\left|x-\delta \right|/\sigma }. \end{array}$$

Correspondingly, the red/blue curve in Figure 2 represents the probability density function of the Laplace/truncated and normalized Laplace distribution, respectively. In the latter one, the output range of the perturbation result is considerably narrowed, and the probability of the output result near the true value remains the maximum. The truncated Laplace distribution curve is not symmetrical like the conventional Laplace distribution under certain parameter conditions, which leads to the deviation between the expected value and the true degree. Nevertheless, because of the limitation of the output range, truncated Laplace has a smaller mean square error than the conventional mechanism under a low privacy budget. Thus, we can infer that the limitation of the length of output interval for user's degree perturbation will greatly affect the utility of the noisy degree vector and the accuracy of the community detection results.

Algorithm 1 describes the local perturbation process in detail. In particular, during each round of user community bipartition, user *i* will receive a bipartite grouping **G** of the user subset **U** including herself from the server for multiple times. Each time, she keeps her personal social data locally and calculates the corresponding true degree vector according to the grouping situation (Line 1). When delivering **G** to the users, the server will notify user *i* of the length of the truncated range, which is denoted as *l*. Based on this, user *i* generates two sequences of output intervals with the same length of truncation corresponding to **U**^1^ and **U**^2^ (Line 2) and obtains the output interval for each dimension according to her true degree vector (Line 3). Afterward, user *i* spends privacy budget *ε* to perturb each dimension. Through the true degree *δ*, the left boundary *L* and the right boundary *R* of the output interval, the scaling parameter *σ*, and the normalization coefficient *n*_*δ*_, as well as the probability density function *p*(*x|x* ∈ [*L*, *R*]), can be calculated (Line 7). To ensure that the perturbation result is meaningful, the output probability of any integer in the output interval is calculated according to the data perturbation steps shown in lines 8–11 to make that any real result obtained by the truncated Laplace mechanism are rounded, which still satisﬁes *ε*-LDP for each query.

### 4.4. Reﬁned EO Algorithm

As mentioned in Section 4.2.1, directly performing the grouping adjustment of the classical EO algorithm and the recalculation of user ﬁtness in the application scenario of LDP will not only cause the excessive allocation of the privacy budget but also lead to a catastrophic increase in the cost of user-server communication. For solving this problem, we attempt to make full use of the uploaded data and propose to form an iterative process inside the server based on the perturbed degree vectors and the corresponding user grouping, thereby greatly reducing the total number of interactions between users and the server during the process of optimal bipartition for each community. Speciﬁcally, we assume that the degree vector of all users remains unchanged after any number of users are migrated to the other group; then, the change in ﬁtness is only related to the calculable *a*_*c*(*i*)_. After the move of user *i*, the sum of user degrees in the original group, where user *i* is located, needs to be subtracted by *i*'s perturbed degree, and the sum of degrees in *i*'s current group needs to be added with *i*'s degree value accordingly. Therefore, the changes of ${\tilde{a}}_{1}$ and ${\tilde{a}}_{2}$ of the two groups can be calculated correspondingly, and the ﬁtness of all users can also be updated according to (3).

In Algorithm 2, we describe the whole process consisting of the server-side migration of users in bipartite grouping and several interactions of degree query to ﬁnally obtain an optimal grouping with converged bipartite modularity.

Initially, after setting the length of the truncated range and the privacy budget of a single query, the server bisects the user subset **U** randomly into two groups (line 1), followed by sending all this information to the users. Each user substitutes the above privacy protection parameters into Algorithm 1 to obtain her perturbed degree vector and uploads it to the center (line 4). After that, the server aggregates these noisy data to directly calculate the total number of edges, ${\tilde{a}}_{1}$ and ${\tilde{a}}_{2}$, the ﬁtness of each user, and the bipartition modularity of the initial grouping (lines 5–10).

Then, the server performs the grouping adjustment step shown in lines 12–21 of Algorithm 2. The user *m*′ with the lowest ﬁtness is migrated to the other group. Based on the noisy degree vector uploaded by users and the ﬁne-tuned ${\tilde{a}}_{1}$ and ${\tilde{a}}_{2}$ after the migration of *m*′ (lines 16–19), the ﬁtness of all users is updated, and the user with the lowest ﬁtness is found out and migrated again. This process will continue to iterate, during which the server only uses the perturbed degree vectors uploaded by users according to the initial random grouping and does not consume any additional privacy budget. From the classical EO algorithm, it can be inferred that the ultimate goal of the iteration is to make the ﬁtness of all users in the ﬁnal convergent grouping situation greater than 0, which means that the bipartition modularity no longer increases. Considering that this situation may not be reached in the end, the convergence conditions are relaxed. When the last two search results are the same user with the minimum ﬁtness (line 14), the migration iteration of users can be stopped for this time.

Considering the degree vector of each user is in fact changing implicitly in the continuous adjustment of user grouping, the iterative migration of users based on the degree vectors of the initial grouping cannot derive the expected near-optimal bipartition of **U**. For this problem, another iterative process of degree query between users and the server is constructed. After the server performs the steps in lines 12–21 based on the initial grouping and the corresponding degree vector, the formed convergent grouping will continue to be delivered to all users as the baseline grouping of a new query to obtain the next new perturbed degree vectors (line 22), and from it, the server will perform the user migration iterative process of the next time (in Algorithm 2, we take *s* to denote the number of degree queries). Therefore, a small iterative migration process is nested in a larger iterative process of degree query. In this way, the number of user interactions with the server and the consumption of the privacy budget can ultimately be greatly reduced. When the algorithm is executed until the bipartition modularity of user convergent grouping **G** does not increase (line 23), the ideal division result of the user subset **U** is obtained.

According to the description of the framework in Section 4.1, when each user subset **U**_*r*−1_ in the initial community division **C**_*r*−1_ of the *r*th round does not increase the total modularity after completing the optimal bipartition, the iteratively splitting process of user subset gets terminated. Therefore, it is necessary to estimate the modularity gain $\Delta \tilde{Q}$ after each execution of Algorithm 2. To obtain unbiased estimation results, each user is required to consume additional privacy budget *ε*_f_ and utilize Laplace mechanism to add calibrated noise on her true degree vector, which is based on the optimal bipartition result of the user subset including her (also as **G**_f_). According to this, the user degree in the subset **U** and the total number of edges $\tilde{L}$ of the user subset can be derived by referring to lines 4 and 5 in Algorithm 2. The total number of edges ${\tilde{L}}_{1}$ and ${\tilde{L}}_{2}$ within each one of the ﬁnal groups can also be directly calculated, i.e.,

(11) $$\begin{array}{c} {\tilde{L}}_{1}=\frac{1}{2}\sum_{j\in U_{\text{f}}^{1}}{\tilde{\delta }}_{j}^{1},\\ {\tilde{L}}_{2}=\frac{1}{2}\sum_{k\in U_{\text{f}}^{2}}{\tilde{\delta }}_{k}^{2}. \end{array}$$

We also use the noisy degree vectors with Laplace noise from the ﬁrst round's optimal bipartition for all users to calculate the total degree of each user and the total number of edges in the entire network. Here, we slightly abuse the notations by using ${\tilde{\delta }}_{i}^{\text{t}}$ and ${\tilde{L}}_{\text{t}}$ to denote them, respectively, and

(12) $$\begin{array}{c} {\tilde{L}}_{\text{t}}=\frac{1}{2}\left(\underset{j\in U_{\text{f}}^{1}}{\sum }{\tilde{\delta }}_{j}^{\text{t}}+\underset{k\in U_{\text{f}}^{2}}{\sum }{\tilde{\delta }}_{k}^{\text{t}}+\underset{i\in U_{0}\backslash U}{\sum }{\tilde{\delta }}_{i}^{\text{t}}\right), \end{array}$$

in which the three items on the right side are mutually independently perturbed with Laplace mechanism. According to the deﬁnition of modularity, the estimated gain of $\tilde{Q}$ after the optimal bipartition of user subset **U** can be calculated, i.e.,

(13) $$\begin{array}{c} \Delta \tilde{Q}=\frac{{\tilde{L}}_{1}}{{\tilde{L}}_{\text{t}}}-{\left(\frac{\sum_{j\in U_{\text{f}}^{1}}{\tilde{\delta }}_{j}^{\text{t}}}{2{\tilde{L}}_{\text{t}}}\right)}^{2}+\frac{{\tilde{L}}_{2}}{{\tilde{L}}_{\text{t}}}-{\left(\frac{\sum_{k\in U_{\text{f}}^{2}}{\tilde{\delta }}_{k}^{\text{t}}}{2{\tilde{L}}_{\text{t}}}\right)}^{2}-\left[\frac{\tilde{L}}{{\tilde{L}}_{\text{t}}}-{\left(\frac{\sum_{j\in U_{\text{f}}^{1}}{\tilde{\delta }}_{j}^{\text{t}}+\sum_{k\in U_{\text{f}}^{2}}{\tilde{\delta }}_{k}^{\text{t}}}{2{\tilde{L}}_{\text{t}}}\right)}^{2}\right],\\ =\frac{\sum_{j\in U_{\text{f}}^{1}}{\tilde{\delta }}_{j}^{\text{t}}\sum_{k\in U_{\text{f}}^{2}}{\tilde{\delta }}_{k}^{\text{t}}-{\tilde{L}}_{\text{t}}\left(\sum_{j\in U_{\text{f}}^{1}}{\tilde{\delta }}_{j}^{2}+\sum_{k\in U_{\text{f}}^{2}}{\tilde{\delta }}_{k}^{1}\right)}{2{\left({\tilde{L}}_{\text{t}}\right)}^{2}}. \end{array}$$

Since each item of equation (13) is an unbiased estimation of its corresponding true value, it can be observed that if its numerator is larger than 0 by the value of its standard deviation, the optimal bipartition will cause positive gain in the total modularity with an adequate probability. Thus, considering that the Laplace noise of ${\tilde{\delta }}_{i}^{\text{t}}$ and ${\tilde{L}}_{\text{t}}$ is independent of that injected to ${\tilde{\delta }}_{j}^{2}$ and ${\tilde{\delta }}_{k}^{1}$ and by using equation (12), we attempt to derive the speciﬁc form of its variance as follows:

(14) $$\begin{array}{c} \text{Var}\left[\underset{j\in U_{f}^{1}}{\sum }{\tilde{\delta }}_{j}^{\text{t}}\underset{k\in U_{f}^{2}}{\sum }{\tilde{\delta }}_{k}^{\text{t}}-{\tilde{L}}_{\text{t}}\left(\underset{j\in U_{f}^{1}}{\sum }{\tilde{\delta }}_{j}^{2}+\underset{k\in U_{f}^{2}}{\sum }{\tilde{\delta }}_{k}^{1}\right)\right]=E\left[{\left(\underset{j\in U_{f}^{1}}{\sum }{\tilde{\delta }}_{j}^{\text{t}}\right)}^{2}\right]E\left[{\left(\underset{k\in U_{f}^{2}}{\sum }{\tilde{\delta }}_{k}^{\text{t}}\right)}^{2}\right]-E\left[2{\tilde{L}}_{\text{t}}\underset{j\in U_{f}^{1}}{\sum }{\tilde{\delta }}_{j}^{\text{t}}\underset{k\in U_{f}^{2}}{\sum }{\tilde{\delta }}_{k}^{\text{t}}\right]\left(\underset{j\in U_{f}^{1}}{\sum }\delta_{j}^{2}+\underset{k\in U_{f}^{2}}{\sum }\delta_{k}^{1}\right)\\ +E\left[{\left({\tilde{L}}_{\text{t}}\right)}^{2}\right]E\left[{\left(\underset{j\in U_{f}^{1}}{\sum }{\tilde{\delta }}_{j}^{2}+\underset{k\in U_{f}^{2}}{\sum }{\tilde{\delta }}_{k}^{1}\right)}^{2}\right]-{\left(\underset{j\in U_{f}^{1}}{\sum }\delta_{j}^{\text{t}}\underset{k\in U_{f}^{2}}{\sum }\delta_{k}^{\text{t}}-L_{\text{t}}\left(\underset{j\in U_{\text{f}}^{1}}{\sum }\delta_{j}^{2}+\underset{k\in U_{f}^{2}}{\sum }\delta_{k}^{1}\right)\right)}^{2}\\ =\text{Var}\left[\underset{j\in U_{f}^{1}}{\sum }{\tilde{\delta }}_{j}^{\text{t}}\right]{\left(\underset{k\in U_{f}^{2}}{\sum }\delta_{k}^{\text{t}}\right)}^{2}+\text{Var}\left[\underset{k\in U_{f}^{2}}{\sum }{\tilde{\delta }}_{k}^{\text{t}}\right]{\left(\underset{j\in U_{f}^{1}}{\sum }\delta_{j}^{\text{t}}\right)}^{2}+\text{Var}\left[\underset{j\in U_{f}^{1}}{\sum }{\tilde{\delta }}_{j}^{\text{t}}\right]\text{Var}\left[\underset{k\in U_{f}^{2}}{\sum }{\tilde{\delta }}_{k}^{\text{t}}\right]\\ -\left(\underset{j\in U_{f}^{1}}{\sum }\delta_{j}^{2}+\underset{k\in U_{f}^{2}}{\sum }\delta_{k}^{1}\right)\left[\text{Var}\left(\underset{j\in U_{f}^{1}}{\sum }{\tilde{\delta }}_{j}^{\text{t}}\right)\underset{k\in U_{f}^{2}}{\sum }\delta_{k}^{\text{t}}+\text{Var}\left(\underset{k\in U_{f}^{2}}{\sum }{\tilde{\delta }}_{k}^{\text{t}}\right)\underset{j\in U_{f}^{1}}{\sum }\delta_{j}^{\text{t}}\right]+\text{Var}\left({\tilde{L}}_{\text{t}}\right)\\ {\left(\underset{j\in U_{f}^{1}}{\sum }\delta_{j}^{2}+\underset{k\in U_{f}^{2}}{\sum }\delta_{k}^{1}\right)}^{2}+{\left(L_{\text{t}}\right)}^{2}\text{Var}\left(\underset{j\in U_{f}^{1}}{\sum }{\tilde{\delta }}_{j}^{2}+\underset{k\in U_{f}^{2}}{\sum }{\tilde{\delta }}_{k}^{1}\right)+\text{Var}\left({\tilde{L}}_{\text{t}}\right)\text{Var}\left(\underset{j\in U_{f}^{1}}{\sum }{\tilde{\delta }}_{j}^{2}+\underset{k\in U_{f}^{2}}{\sum }{\tilde{\delta }}_{k}^{1}\right). \end{array}$$

Noticing that the variance of Laplace noise with privacy budget *ε* and sensitivity Δ*f* is 2(Δ*f*/*ε*)^2^, we can solve all the variance item in equation (14) and reach the final expression as

(15) $$\begin{array}{c} \text{Var}\left[\underset{j\in U_{\text{f}}^{1}}{\sum }{\tilde{\delta }}_{j}^{\text{t}}\underset{k\in U_{\text{f}}^{2}}{\sum }{\tilde{\delta }}_{k}^{\text{t}}-{\tilde{L}}_{\text{t}}\left(\underset{j\in U_{\text{f}}^{1}}{\sum }{\tilde{\delta }}_{j}^{2}+\underset{k\in U_{\text{f}}^{2}}{\sum }{\tilde{\delta }}_{k}^{1}\right)\right]=\frac{4\left|U_{\text{f}}^{1}\right|}{\varepsilon_{\text{f}}^{2}}{\left(\underset{k\in U_{\text{f}}^{2}}{\sum }\delta_{k}^{\text{t}}\right)}^{2}+\frac{4\left|U_{\text{f}}^{2}\right|}{\varepsilon_{\text{f}}^{2}}{\left(\underset{j\in U_{\text{f}}^{1}}{\sum }\delta_{j}^{\text{t}}\right)}^{2}+\frac{16\left|U_{\text{f}}^{1}\right|\left|U_{\text{f}}^{2}\right|}{\varepsilon_{\text{f}}^{4}}-\left(\underset{j\in U_{\text{f}}^{1}}{\sum }\delta_{j}^{2}+\underset{k\in U_{\text{f}}^{2}}{\sum }\delta_{k}^{1}\right)\\ \left[\frac{4\left|U_{\text{f}}^{1}\right|}{\varepsilon_{\text{f}}^{2}}\underset{k\in U_{\text{f}}^{2}}{\sum }\delta_{k}^{\text{t}}+\frac{4\left|U_{\text{f}}^{2}\right|}{\varepsilon_{\text{f}}^{2}}\underset{j\in U_{\text{f}}^{1}}{\sum }\delta_{j}^{\text{t}}\right]+\frac{N}{\varepsilon_{\text{f}}^{2}}{\left(\underset{j\in U_{\text{f}}^{1}}{\sum }\delta_{j}^{2}+\underset{k\in U_{\text{f}}^{2}}{\sum }\delta_{k}^{1}\right)}^{2}+\frac{2\left|U\right|{\left(L_{\text{t}}\right)}^{2}}{\varepsilon_{\text{f}}^{2}}+\frac{2N\left|U\right|}{\varepsilon_{\text{f}}^{4}}. \end{array}$$

After the server obtains the optimal bipartition of **U** and receives corresponding noisy degree vector from each user in **U**, also with their noisy total degrees as well as the total edges, we can simply calculate the estimation value of equation (13) numerator. If it is greater than the square root of the right side of equation (15), the modularity gain caused by **U**'s division is considered positive, and the bipartition {**U**_f_^1^, **U**_f_^2^} will be accepted to replace {**U**} in the community division of the entire graph.

### 4.5. Theoretical Analysis

#### 4.5.1. Proof of *ε*-LDP Guarantee

This section will prove that the truncated Laplace mechanism proposed in Section 4.3 satisﬁes *ε*-LDP.

In the process of degree perturbation by users, it is assumed that the local relationship data of each user in two adjacent graphs (differed by any single edge) are *D*_1_ and *D*_2_, which are either the same or different by one bit. The function *f*(*D*) is the degree query function. Therefore, the local sensitivity is $\Delta f=\underset{D_{1},D_{2}}{\max}\left|f\left(D_{1}\right)-f\left(D_{2}\right)\right|=1$. According to the step of truncated interval selection, it is assumed that both *f*(*D*_1_) and *f*(*D*_2_) are in the interval [*L*, *R*]; thus, any result *s* ∈ **ℝ** obtained by the perturbation also satisﬁes *s* ∈ [*L*, *R*]. The truncated Laplace mechanism is set to be *M*. According to Deﬁnition 1, it is necessary to prove that the following inequality is always valid:

(16) $$\begin{array}{c} e^{-\varepsilon }\leq \frac{\mathrm{Pr}\left[M\left(f\left(D_{1}\right)\right)=s\right]}{\mathrm{Pr}\left[M\left(f\left(D_{2}\right)\right)=s\right]}\leq e^{\varepsilon }. \end{array}$$

**Figure 3:**
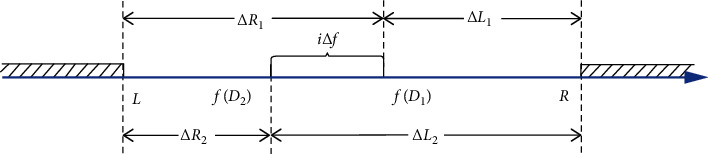
The diagram of the truncated range for degree perturbation.

Without loss of generality, as shown in Figure 3, it is assumed that *L* ≤ *f*(*D*_2_) ≤ *f*(*D*_1_) ≤ *R*, and let Δ*L*_1_=|*f*(*D*_1_) − *R*|, Δ*R*_1_=|*f*(*D*_1_) − *L*|, Δ*L*_2_=|*f*(*D*_2_) − *R*|, Δ*R*_2_=|*f*(*D*_2_) − *L*|, and |*f*(*D*_1_) − *f*(*D*_2_)|=*i*Δ*f*(*i*=0,1). The following theorems are given and proved below.

**Theorem 1 .**

With the given truncated interval [*L*, *R*] and the privacy budget *ε*, as well as the user's local degree *f*(*D*_1_) or *f*(*D*_2_), if the scaling parameter *σ* in the Laplace distribution satisﬁes *σ*=2Δ*f*/*ε*, the results obtained by perturbing the true degrees *f*(*D*_1_) or *f*(*D*_2_) according to the probability distribution of equation (10) must satisfy *ε*-LDP.

**Proof**

Replace the probability expression in equation (16) with equation (10). Then, replace *I*_*L*2_ and *I*_*R*2_ with *I*_*L*1_ and *I*_*R*1_, and use the triangle inequality, and we can obtain

(17) $$\begin{array}{c} \frac{n_{\delta 1}/2\sigma \cdot e^{-\left|s-f\left(D_{1}\right)\right|/\sigma }}{n_{\delta 2}/2\sigma \cdot e^{-\left|s-f\left(D_{2}\right)\right|/\sigma }}\leq \frac{1-\left(I_{L1}e^{i\Delta f/\sigma }+I_{R1}e^{-i\Delta f/\sigma }\right)}{1-I_{L1}-I_{R1}}e^{i\Delta f/\sigma }. \end{array}$$

To prove that the left side is smaller than *e*^*ε*^, it is attempted to prove that the right side does not exceed *e*^*iε*^ on *i* ∈ [0,1]. Thus, taking *i* ∈ [0,1] as the independent variable, let

(18) $$\begin{array}{c} F\left(i\right)=\frac{1-\left(I_{L1}e^{i\Delta f/\sigma }+I_{R1}e^{-i\Delta f/\sigma }\right)}{1-I_{L1}-I_{R1}},\\ R\left(i\right)=e^{i\left(\varepsilon -\Delta f/\sigma \right)}. \end{array}$$

By careful observation, when *i*=0, *F*(*i*)=*R*(*i*)=1. If let *F*(*i*) ≤ *R*(*i*), when *i* ≥ 0, we should require that *F*′(0) ≤ *R*′(0). By solving the inequality, the value range of *σ* is obtained, i.e.,

(19) $$\begin{array}{c} \sigma \geq \frac{\Delta f}{\varepsilon }\frac{2I_{L1}-1}{I_{L1}+I_{R1}-1}. \end{array}$$

In order to ensure that *F*′(*i*) does not exceed *R*′(*i*) when *i* > 0, the secondary derivatives of these two functions are calculated as follows:

(20) $$\begin{array}{c} F^{\prime \prime }\left(i\right)=-{\left(\frac{\Delta f}{\sigma }\right)}^{2}\frac{I_{R1}e^{-i\Delta f/\sigma }+I_{L1}e^{i\Delta f/\sigma }}{1-I_{L1}-I_{R1}},\\ R^{\prime \prime }\left(i\right)={\left(\varepsilon -\frac{\Delta f}{\sigma }\right)}^{2}e^{i\left(\varepsilon -\Delta f/\sigma \right)}. \end{array}$$

According to equation (8) and equation (9), it can be known that *I*_*L*1_+*I*_*R*1_ < 1 is always valid. Therefore, when *i* > 0, we can infer that *F*^″^(*i*) < 0 and *R*^″^(*i*) ≥ 0, which means that *F*′(*i*) is monotonically decreasing and *R*′(*i*) is monotonically increasing. Because *σ* satisﬁes equation (19) to ensure that *F*′(0) ≤ *R*′(0), *F*′(*i*) ≤ *R*′(*i*) is always true for *i* ≥ 0. Thus, it can be inferred that *F*(*i*) ≤ *R*(*i*) always holds on *i* ∈ [0,1], and accordingly,

(21) $$\begin{array}{c} \frac{\mathrm{Pr}\left[M\left(f\left(D_{1}\right)\right)=s\right]}{\mathrm{Pr}\left[M\left(f\left(D_{2}\right)\right)=s\right]}=\frac{n_{\delta 1}/2\sigma \cdot e^{-\left|s-f\left(D_{1}\right)\right|/\sigma }}{n_{\delta 2}/2\sigma \cdot e^{-\left|s-f\left(D_{2}\right)\right|/\sigma }}\leq e^{i\varepsilon }\leq e^{\varepsilon }. \end{array}$$

Similarly, the above deducing process can also be used to prove the validity of the left half of equation (16) under the following condition:

(22) $$\begin{array}{c} \sigma \geq \frac{\Delta f}{\varepsilon }\frac{2I_{R2}-1}{I_{L2}+I_{R2}-1}. \end{array}$$

Therefore, if truncated Laplace mechanism is to strictly satisfy *ε*-LDP, *σ* must simultaneously satisfy

(23) $$\begin{array}{c} \sigma \geq \frac{\Delta f}{\varepsilon }\frac{2I_{L1}-1}{I_{L1}+I_{R1}-1}=\frac{2\Delta f}{\varepsilon }\frac{1}{1+\left(1-e^{-\Delta L_{1}/\sigma }\right)/\left(1-e^{-\Delta R_{1}/\sigma }\right)},\\ \sigma \geq \frac{\Delta f}{\varepsilon }\frac{2I_{R2}-1}{I_{L2}+I_{R2}-1}=\frac{2\Delta f}{\varepsilon }\frac{1}{1+\left(1-e^{-\Delta R_{2}/\sigma }\right)/\left(1-e^{-\Delta L_{2}/\sigma }\right)}. \end{array}$$

Finally, extreme cases are used to ﬁnd the strict lower bound of *σ*, which happens when Δ*L*_1_=0 or Δ*R*_2_=0, and accordingly,

(24) $$\begin{array}{c} \sigma \geq \frac{2\Delta f}{\varepsilon }. \end{array}$$

Therefore, if *σ* satisﬁes equation (24), it must also satisfy equation (23). The conclusion is thus proved.

## 5. Experiments

In this section, the experiments are performed to evaluate our proposed method.

### 5.1. Experimental Methods

#### 5.1.1. Datasets

Two datasets published in the Stanford Network Analysis Project (SNAP) are used to conduct the experiments:

1. Facebook dataset [37]: this dataset contains 4039 Facebook users and 88234 relationship edges (undirected edges) formed among them. In addition, this dataset is one of the classic datasets employed for complex network community detection.
2. Facebook page network dataset about the government [38]: this dataset involves 7057 web pages about government information, and the edges between the web pages represent their mutual likes (undirected edges). After removing the self-loop edges in the original data, the total number of edges in the network is 89429.

#### 5.1.2. Compared Methods

In order to evaluate the performance of LDPCD, the proposed method is compared with the following two methods.

1. LDPGen [19]: with this method, a synthetic private social graph under LDP protection is generated. Besides, the experiment in our paper still follows the parameter settings of LDPGen, in which two times of queries on user degree vectors and user clustering based on the k-means method in terms of degree vectors are implemented, and the total privacy budget is evenly distributed. Then, based on the generated synthetic social graph, the Louvain community detection algorithm is ﬁnally adopted.
2. LFGDPR [20]: using this method, a variety of statistical graph metrics, including clustering coefficient distribution and subgraph modularity, are estimated under LDP. In accordance with all its technical details, the experiment in our paper optimally allocates the total privacy budget and then perturbs the user's total degree and adjacency bit vector, respectively. Furthermore, according to the proposed Louvain community detection algorithm based on LFGDPR, the network community is divided.

#### 5.1.3. Utility Metrics

According to Section 3.4, the speciﬁc meanings of the three utility metrics, namely, modularity, ARI (adjusted random index), and AMI (adjusted mutual information), are elaborated.

Modularity: the function of modu(**C**) is to calculate the total modularity of a community division result **C** based on the original real network. Therefore, modu(**C**_p_) is taken as the evaluation index for the quality of community detection results under privacy protection, with the value range of [−1/2, 1].

ARI and AMI: given all users {*u*_1_,…, *u*_*N*_} and their two grouping conditions *X*={*x*_1_,…, *x*_*u*_} and *Y*={*y*_1_,…, *y*_*v*_}, *n*_*ij*_ represents the number of users shared by group *x*_*i*_ and group *y*_*j*_, that is, *n*_*ij*_=|*X*_*i*_∩*Y*_*j*_|, and 1 ≤ *i* ≤ *u* and 1 ≤ *j* ≤ *v*. In addition, let *a*_*i*_=∑_*j*_*n*_*ij*_ and *b*_*j*_=∑_*i*_*n*_*ij*_, and deﬁne the index to measure the similarity between groupings *X* and *Y* as

(25) $$\begin{array}{c} \text{ARI}\left(X,Y\right)=\frac{\sum_{ij}\left(\begin{array}{c} n_{ij}\\ 2 \end{array}\right)-\left[\sum_{i}\left(\begin{array}{c} a_{i}\\ 2 \end{array}\right)\sum_{j}\left(\begin{array}{c} b_{j}\\ 2 \end{array}\right)\right]/\left(\begin{array}{c} N\\ 2 \end{array}\right)}{1/2\left[\sum_{i}\left(\begin{array}{c} a_{i}\\ 2 \end{array}\right)+\sum_{j}\left(\begin{array}{c} b_{j}\\ 2 \end{array}\right)\right]-\left[\sum_{i}\left(\begin{array}{c} a_{i}\\ 2 \end{array}\right)\sum_{j}\left(\begin{array}{c} b_{j}\\ 2 \end{array}\right)\right]/\left(\begin{array}{c} N\\ 2 \end{array}\right)},\\ \text{AMI}\left(X,Y\right)=\sum_{i=1}^{u}\sum_{j=1}^{v}\sum_{n_{ij}={\left(a_{i}+b_{j}-N\right)}^{+}}^{\min\left(a_{i},b_{j}\right)}\frac{n_{ij}}{N}\log\left(\frac{N\cdot n_{ij}}{a_{i}b_{j}}\right)\times \frac{a_{i}!b_{j}!\left(N-a_{i}\right)!\left(N-b_{j}\right)!}{N!n_{ij}!\left(a_{i}-n_{ij}\right)!\left(b_{j}-n_{ij}\right)!\left(N-a_{i}-b_{j}+n_{ij}\right)!}. \end{array}$$

Among them, (*a*_*i*_+*b*_*j*_ − *N*)^+^ means max(1, *a*_*i*_+*b*_*j*_ − *N*). Speciﬁcally, ARI represents the frequency of agreements between the two obtained groupings over all element pairs, and AMI quantitatively refers to the amount of information shared by the two groupings *X* and *Y*. In the case of the same grouping, AMI is usually higher than ARI, both with the value range of [0,1]. Higher values of ARI and AMI mean that two groupings of the same user set are more similar. Therefore, in the experiment, higher ARI(**C**_p_, **C**_t_) or AMI(**C**_p_, **C**_t_) indicates that the community detection result under LDP has higher accuracy.

#### 5.1.4. Parameter Settings

In this paper, the parameter settings of the LDP-based community detection algorithm are as follows.

As shown in Algorithm 2, each time the server queries user degree, the assigned privacy budget is equal, which is denoted as *ε*. Other than that, calculating the total modularity gain also requires to be allocated a certain privacy budget *ε*_f_ so as to query the degree vector perturbed by Laplace mechanism under the ﬁnal bipartition **G**_f_. Thus, we set the value range of *ε*_f_ within 0.02 to 0.1, while in contrast, that of *ε* is from 0 to 0.05. If the total number of rounds for the division of user communities is *r* and the number of times that user *i* uploads data to the server in each round of bipartite division is listed as {*s*_1_^*i*^, *s*_2_^*i*^,…, *s*_*r*_^*i*^}, the total privacy cost is $\varepsilon_{\text{total}}=\underset{j\in \left[0,N-1\right]}{\max}\left(\varepsilon \sum_{n=1}^{r}s_{n}^{j}+r\varepsilon_{f}\right)$.

Besides, in the truncated Laplace mechanism, the length of truncated range *l* is one of the key parameters that needs to be explored. On the one hand, the degree distribution of complex social networks is usually expressed as power-law distribution [29], in which the total degree of most users is below the average. Therefore, in this experiment, the maximum value of *l* for the Facebook dataset (average degree of 42) and the Government dataset (average degree of 25) are set to 30 and 20, respectively. On the other hand, excessively small *l* will cause the perturbed degree to be too close to the true one, which can lead to the privacy disclosure. Considering that, the minimum value of *l* is set to 5.

In terms of the total privacy budget/cost, considering when the value of it is greater than 2.5 in [20], the probability of each bit in the adjacency vector not to be ﬂipped exceeds 90%, which will expose the privacy concerning most of the user's connected edges (for the Facebook dataset, the allocated privacy budget for perturbing user's bit vector is 2.225. Since the ratio of bit flipping probability to the unflipping probability is *e*^−2.225^ according to the definition of LDP, we can easily understand that the perturbed bit remains unchanged with a probability of *e*^2.225^/(1+*e*^2.225^)=0.902472). In this case, in order to ensure an appropriate protection strength of LDP, the total privacy budget in the contrast experiments is limited to 0.1–2.5.

#### 5.1.5. Experimental Setting

Based on the given total privacy budget *ε*_total_, LDPGen and LFGDPR are separately tested 100 times in our experiment. Then, the average values of modularity, ARI and AMI, from all community detection results are taken as the ﬁnal result. While for LDPCD, the average result of the three measurement metrics obtained by 100 times of experiments with their actual total privacy cost in the range of *ε*_total_ ± 0.05 is considered as the ﬁnal result of our method.

### 5.2. Experimental Results

In this part of the paper, we will present detailed experimental results and corresponding analysis to demonstrate the feasibility of our proposed method. Firstly, some general but necessary data are enumerated, which give a brief outline of the comparison between the classical EO algorithm and LDPCD when they are separately employed in the datasets. Then, deeper discussions about the influence of parameters and the contrast result of LDPCD with two state-of-the-art methods will be elaborated according to the figures.

**Figure 4:**
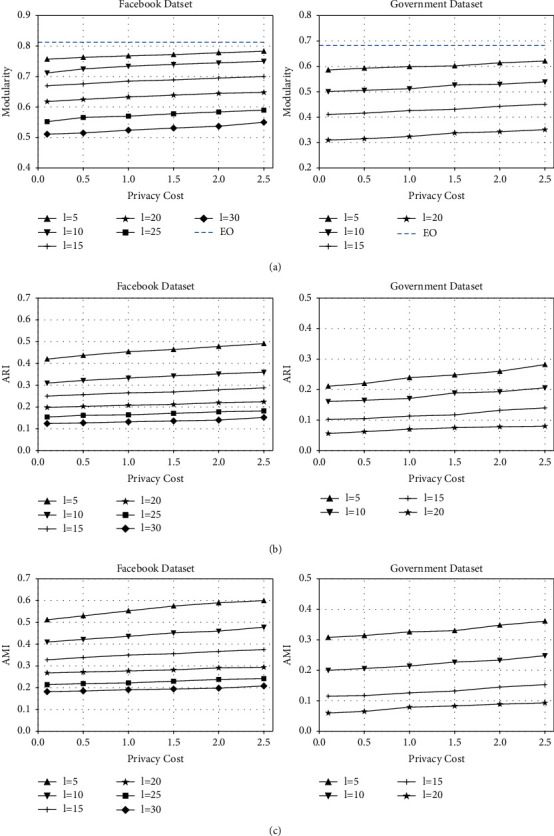
The experimental results of LDPCD on the Facebook and Government datasets (the bold dashed lines in Figure 4(a) indicate that the modularity result based on nonprivate EO algorithm in the Facebook/Government dataset is 0.813/0.682, respectively). (a) Modularity. (b) ARI. (c) AMI.

For the Facebook (Government) dataset, although the average modularity of the community partition results by classical EO algorithm reaches up to 0.813 (0.682) (see Figure 4(b)), the total times of user migration and recalculation of degree vectors exceed 20000 (35000). In contrast, under different parameter settings of LDPCD, the average modularity is within the range of 0.51–0.79 (0.31–0.63), with the total times of degree queries from 25 to 50 (from 40 to 70). In terms of the community numbers, the EO algorithm finally outputs 15 (29) user subsets as the ground truth, while LDPCD partitions all users into 8–16 (15–32) groups. From the abovementioned data, it can be obviously observed that our method simplifies the implementation of EO community divisive algorithm to a considerable extent and significantly reduce the communication cost of user-server interactions, which, in the same time, obtains utility-guaranteed results similar to the ground truth.

#### 5.2.1. Questions about the Experiment

In the following sections, the effectiveness of LDPCD is veriﬁed based on experimental results. Besides, through the results, the following two questions are answered:

1. EQ **1**: how do the length of truncated range and the total privacy cost affect the accuracy of community detection of LDPCD separately?
2. EQ **2**: what are the advantages of the proposed method LDPCD compared with the existing methods under privacy protection of the same strength?

#### 5.2.2. Inﬂuence of Experimental Parameters on the Results

From Figure 4, it can be seen that the length of the truncated output range has a more signiﬁcant impact on the community division result than the total privacy cost. With the same privacy cost in the Facebook dataset (Government dataset), the average value concerning maximal changes of modularity, ARI, and AMI under the inﬂuence of *l* is 0.242, 0.322, and 0.367 (0.272, 0.173, and 0.253); while under the same output interval length, the average value of maximal changes in modularity, ARI, and AMI under the inﬂuence of total privacy cost *ε*_total_ is 0.034, 0.040, and 0.047 (0.039, 0.045, and 0.043), as shown in Figures 4(a), 4(b), and 4(c), respectively. For these observed results, there are two main reasons. Firstly, with the introduction of the truncated Laplace mechanism, the variance of degree and the expected error of ﬁtness $\tilde{\lambda }$ are limited and closely related to *l*. Besides, the smaller the *l*, the smaller the perturbation noise of the degree vector and the total degree. If the true degree is large enough when compared with *l*, the deviation of ﬁtness $\tilde{\lambda }$ relative to its true value will be signiﬁcantly reduced, and the probability of incorrect migration of the user during grouping adjustment will also decrease. As a result, the utility of community division will improve to a great extent with the decrease of *l*. Secondly, since multiple rounds of bipartition and several times of degree query in each round will consume privacy budget; even if the ﬁnal total privacy cost experiences obvious increase, the privacy budget allocated for a single query remains relatively limited. Thus, the accuracy of the perturbation result by truncated Laplace mechanism is slightly enhanced (as shown in Table 2). In this case, the accuracy of community detection increases steadily with the rise of total privacy cost.

Moreover, by comparing the graphs in Figures 4(a), 4(b), and 4(c) separately, it can be seen that, under the same *l* and *ε*_total_, the community detection results of the Facebook dataset are more similar to their ground truth than the Government dataset. The main reason is that the average degree of users in the Facebook dataset is higher. With similar total number of edges, the user nodes in the Government dataset is 1.75 times that of the Facebook dataset and its network structure is sparser; thus, the nodes with small total degree account for a larger proportion in it (for example, the ratio of nodes with their degree below 5 and 10 in the Facebook/Government dataset are 0.113 and 0.238/0.232 and 0.398, respectively). Therefore, under the same setting of *l*, more nodes in the Government dataset will be distributed in the output interval very close to [0, *l*]. Meanwhile, we note that the closer the output interval to [0, *l*] is, the greater the deviation of the noisy ﬁtness $\tilde{\lambda }$ from its true value will be, resulting in a larger probability of incorrect node migration during the grouping adjustment. In that case, under the same privacy parameter settings, the utility of the community division result of the Government dataset will decrease more than that of the Facebook dataset.

#### 5.2.3. Comparison with the State-of-the-Art Methods

**Figure 5:**
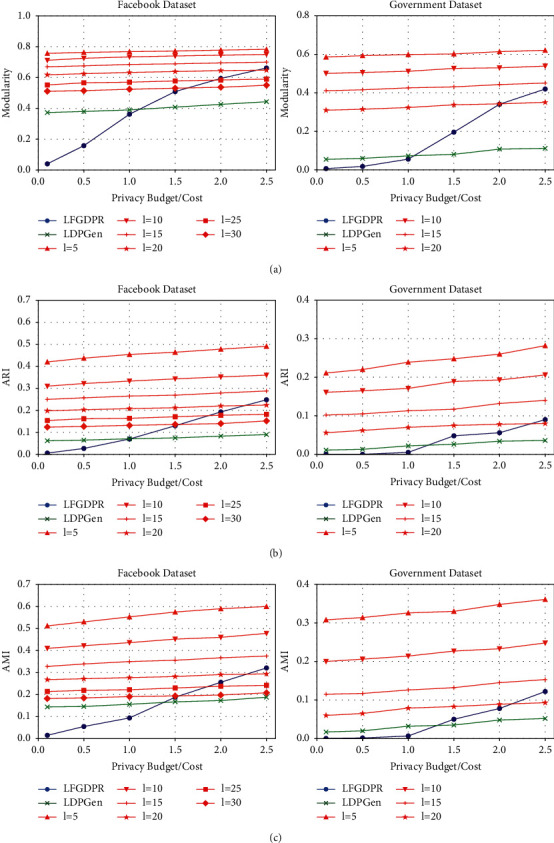
The results obtained from the comparative experiment of LDPGen, LFGDPR, and LDPCD. (a) Modularity. (b) ARI. (c) AMI.

In this section, the experimental results of our proposed method are compared with those of the classical methods, namely, LDPGen [19] and LFGDPR [20]. The contrast results are shown in Figure 5.

Through a comprehensive analysis in Figure 5, it can be found that the result of LDPCD under all settings of *l* is better than that of the LDPGen method under the same total privacy budget/cost. The similarity between LDPGen and our method is that both enquire the user degree vector in a certain grouping situation, and the processed relationship data are all coarse-grained statistics, thus inevitably leading to the loss of some local information in the original social graph. Whereas, the difference is that LDPGen clusters users from the perspective of the similarity of the degree vector instead of their contribution to the total modularity, and based on the ﬁnal grouping, a synthetic social graph is generated by the Chung-Lu probability model for further analysis of community structure. However, the user clustering and graph generation methods result in low utility of the community division under LDP, for the Chung-Lu model randomly connects user node pairs in the same group or different groups, which weakens the distinctiveness of densely connected node clusters in the original network and destroys the community features. This can be observed in Figures 5(b) and 5(c) that with the increase of the total privacy budget, even if the accuracy of the degree vector is gradually improved, the utility of the community detection result on the synthetic social graph is still in a low state and not signiﬁcantly enhanced. In this case, it indicates that LDPGen cannot well balance the relationship between the strength of privacy protection and the accuracy of community mining results. In contrast, by using LDPCD, the user's contribution to the total modularity is calculated with the degree vector, and the community structure of the original network is gradually restored through multiple times of degree query and grouping adjustment. Otherwise, as mentioned in Section 5.2.2 that *l* plays a leading role in the experimental results, the utility of the community division obtained by LDPCD can maintain a relatively high level under different total privacy costs.

For LFGDPR, the perturbation object is the user's total degree and the most ﬁne-grained adjacent bit. Since the processed data involve sensitive neighboring information, the privacy budget in the LFGDPR method should be adjusted to a small value to satisfy the requirement of sufficient privacy protection strength. As shown in Figures 5(a), 5(b), and 5(c), when the total privacy budget/cost of the Facebook dataset and the Government dataset is below 1.5 and 2.0, respectively, the accuracy of the community detection results given by LDPCD is higher than that of the LFGDPR method under all settings of *l*. Especially, when *ε*_total_ is less than 1.0 and the privacy protection is strong enough, LDPCD has obvious advantages over LFGDPR. When *ε*_total_=1, the worst ARI/AMI value of the community detection result of the Facebook (Government) dataset of our proposed method is 1.89/2.03 (14.00/11.97) times that of LFGDPR (see Figures 5(b) and 5(c)), which also reﬂects that the LFGDPR method with low privacy budget has unsatisfactory effect on community detection in sparser social networks. When the privacy budget/cost is higher (*ε*_total_ > 2.0), the utility of the community detection result by LFGDPR is slightly better than that of some groups of experiments with larger *l*. However, we should note that when LFGDPR is adopted in the Facebook dataset (the Government dataset) with *ε*_total_=2.0, the probability of a single bit remaining unﬂipped is 5.81 (5.26) times than that of being ﬂipped, respectively. This means LFGDPR is more liable to expose privacy under such privacy parameter settings. Thus, in contrast, LDPCD is superior to the LFGDPR method in terms of both the utility of the community detection results and the strength of privacy protection.

Furthermore, it should be emphasized that although the community detection accuracy of LDPCD improves slightly with the increase of the total privacy cost under a ﬁxed truncated interval length *l*, in practical applications, appropriately increasing *l* can be considered when *ε*_total_ is low while reducing *l* can be taken into account when *ε*_total_ is high, so as to achieve a better tradeoff between the strength of privacy protection and the quality of community division results.

## 6. Conclusion

In this paper, LDPCD, a novel community detection method, is proposed based on the local differential privacy model. In the framework of LDPCD, the truncated Laplace mechanism with local differential privacy is employed to enhance the accuracy of user perturbation data. Other than that, by reﬁning the community divisive algorithm based on extremal optimization, the number of interactions between users and the server is reduced, thus reducing the total privacy cost and ensuring strong privacy protection. Based on the above data perturbation and community detection algorithms, the community detection results with high utility are ﬁnally obtained. Furthermore, according to the experimental results on two real-world datasets, it can be concluded that LDPCD has the same or higher accuracy of community detection compared with the state-of-the-art methods under different settings of privacy protection parameters. In addition, LDPCD is featured with obvious superiority under strong privacy protection.

## Figures and Tables

**Algorithm 1:**
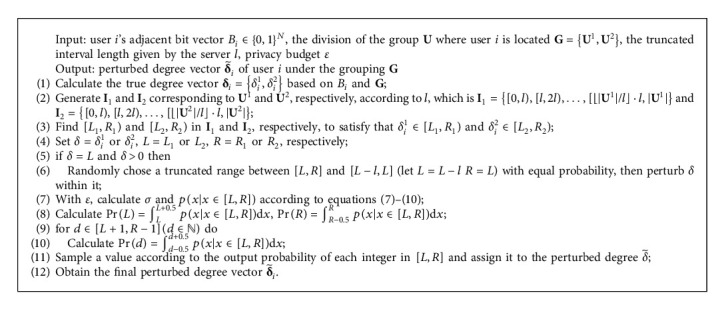
The implementation of truncated Laplace mechanism on the user side

**Algorithm 2:**
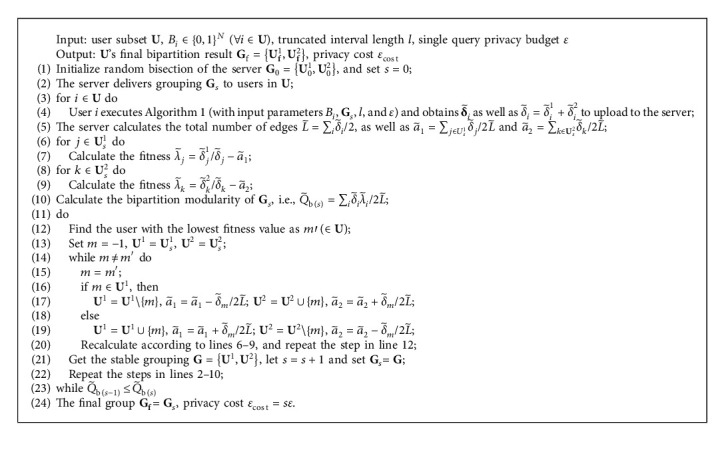
User's group bipartition by reﬁned EO.

**Table 1:** The description of the main notations used in this article.

Symbols	Descriptions
**U**_0_	The set of all users
**U**	A user subset of **U**_0_
**G**	A bipartition of **U**
$\lambda_{i}/{\tilde{\lambda }}_{i}$	The true/noisy ﬁtness of user *i* in terms of **G**
$\delta_{i}/{\tilde{\delta }}_{i}$	*i*'s true/noisy degree in **G**
$\delta_{i}/{\tilde{\delta }}_{i}$	*i*'s true/noisy degree vector in **G**
$\tilde{Q}$	The estimated modularity of a community division of all users
${\tilde{Q}}_{b}$	The estimated bipartition modularity of **G**
**G**_f_	The ﬁnal bipartite grouping of **U** with converged ${\tilde{Q}}_{b}$
**C**_*r*_	The community division result of all users of the *r*th round bipartition
**U**_*r*_	A certain user subset (community) of **C**_*r*_
$\Delta \tilde{Q}$	The gain of $\tilde{Q}$ caused by the substitution of **G**_f_ for {**U**} in user community division
*ε*	The privacy budget used for each query on user's degree vector based on **G**
*ε*_f_	The privacy budget used for the query on user's degree vector based on **G**_f_ to estimate $\Delta \tilde{Q}$

**Table 2:** The standard deviation of the noise of the truncated Laplace mechanism under different parameter settings of the Facebook dataset.

-		*l*=5	*l*=10	*l*=15	*l*=20	*l*=25	*l*=30
*ε*_total_=0.1	*ε*=0.0002	2.166	4.160	6.152	8.144	10.136	12.127
*ε*_total_=0.5	*ε*=0.007	2.161	4.144	6.117	8.082	10.038	11.987
*ε*_total_=1.0	*ε*=0.016	2.156	4.122	6.070	7.998	9.909	11.802
*ε*_total_=1.5	*ε*=0.02	2.153	4.113	6.049	7.962	9.852	11.719
*ε*_total_=2.0	*ε*=0.03	2.147	4.082	5.996	7.869	9.708	11.514
*ε*_total_=2.5	*ε*=0.04	2.141	4.065	5.944	7.777	9.565	11.308

## Data Availability

The experimental data used to support the findings of this study are available upon request to the author.
